# Biodegradable Polymer-Coated Surgical Sutures for Controlled and Sustained Release of Sirolimus, Tacrolimus, and Paclitaxel

**DOI:** 10.3390/ijms27083695

**Published:** 2026-04-21

**Authors:** Joanna Chałupka, Karolina Piecyk, Karol Kurpiejewski, Adam Sikora

**Affiliations:** 1 Department of Pharmaceutical Technology, Faculty of Pharmacy, Medical Biotechnology and Laboratory Medicine, Pomeranian Medical University in Szczecin, 71-251 Szczecin, Poland; joanna.chalupka@pum.edu.pl; 2 Department of Medicinal Chemistry, Faculty of Pharmacy, Collegium Medicum in Bydgoszcz, Nicolaus Copernicus University in Toruń, Dr. A. Jurasza 2, 85-089 Bydgoszcz, Poland; 3 Faculty of Chemistry, University of Warsaw, Pasteura 1, 02-093 Warszawa, Poland

**Keywords:** drug-eluting sutures, sirolimus, tacrolimus, paclitaxel, controlled drug release, biodegradable polymers

## Abstract

Biodegradable polymer-coated surgical sutures represent a promising strategy for localized drug delivery to prevent post-surgical complications, such as restenosis, inflammation, and excessive tissue proliferation. In this study, bioactive coatings based on poly(L-lactic acid) (PLA), polycaprolactone (PCL), chitosan, and their binary blends were developed and applied to PLA-based surgical sutures for controlled release of sirolimus, tacrolimus, and paclitaxel. A total of 36 coated suture formulations were prepared using solvent-based deposition techniques and systematically evaluated. In vitro drug release studies conducted under physiological conditions (PBS, 37 °C) over a 12-week period demonstrated sustained and formulation-dependent release profiles. Cumulative drug release varied significantly depending on polymer composition, ranging from 17.53% to 90.93% for sirolimus, 70.93% to 98.50% for tacrolimus, and 34.62% to 67.65% for paclitaxel. PLA-based coatings generally exhibited faster release kinetics, whereas PCL-containing formulations showed slower, more sustained release. Binary polymer blends enabled fine-tuning of release profiles, demonstrating tunable drug delivery performance. All coatings maintained structural integrity during handling and simulated suturing conditions. These findings confirm that polymer composition plays a critical role in controlling drug release kinetics and demonstrate the feasibility of biodegradable polymer-coated sutures as a versatile platform for sustained, localized drug delivery in surgical and vascular applications.

## 1. Introduction

Biocompatible and biodegradable polymers become increasingly important in bio-medical engineering, particularly in devices for temporary wound closure and implanta-ble medical devices. Among the most popular are synthetic polymers: polycaprolactone (PCL) and polylactic acid (PLA) [1,2]. PCL and PLA are highly processable, flexible and can find wide applicability. PLA, PCL, and chitosan are widely recognized biodegradable and biocompatible polymers commonly used in biomedical applications, where PCL finds application in tissue engineering, drug release, and as a scaffold material due to its biocompatibility and rate-controlled biodegradation [3,4]. However, its low degradation rate could be a setback in certain applications, resulting in the synthesis of PCL-based composites and copolymers to enhance its properties [3,5]. On the other hand, PLA has attracted immense attention owing to its beneficial properties, including high mechanical strength, reproducible degradation rate, and nontoxic metabolic by-products [6]. Upon hydrolysis, PLA degrades to carbon dioxide and water through an intermediate of lactic acid, a natural metabolite in human physiology [6]. All these attributes have led to its widespread use in surgery, including absorbable sutures, orthopedic fixation hardware, and tissue scaffolds [2,6]. PLA is more brittle and degrades faster than PCL with lower surface energy, leading to lower cell adhesion [5,7]. Another exciting possibility that has been studied in the recent past is also a combination of PCL and PLA (PLA/PCL blend) [5]. It was shown that the combination of PLA and PCL could deliver composite materials that had the benefit of both the polymers, enhancing mechanical properties and rates of degradation. Despite these advantages, PLA/PCL-based sutures rank among the clinical challenges, of which one important one is postoperative infections of the surgical site (SSI) [8]. Traditional approaches are largely derived from systemic antibiotic delivery, either oral or intravenous. Systemic delivery is, however, accompanied by several drawbacks like limited bioavailability, toxicity, and increasing prospect of antimicrobial resistance [8,9]. These limitations highlight the need for new, innovative products that will locally deliver therapeutic drugs, to the site-specific wound interface.

Drug-eluting sutures have emerged as a potential solution to address these issues, which can deliver localized pharmacologic effects while minimizing systemic exposure. Different methods have been explored for the deposition of bioactive compounds into or onto sutures, such as deposition into biodegradable microspheres, immersion in a drug solution, and electrospinning techniques [9,10,11]. While these methods demonstrated proof of concept, they frequently suffer from poor reproducibility, low drug loading efficiency and, most importantly, limited control over release kinetics. Achieving reproducible and controlled drug release remains an innate challenge, as wound healing drug therapy is extremely time-dependent in its therapeutic window. Early depletion of the drug with diminished clinical effectiveness occurs with sustained burst release, whereas slow release at supratherapeutic levels leads to local toxicity and prevents tissue regeneration [12]. Drug-eluting suture design thus requires a subtle balance between drug stability, polymer degradation kinetics, and tissue-specific therapeutic demands. Recent developments in polymer chemistry and nanotechnology have facilitated the opening up of new prospects for the engineering of bioactive coatings that can be formulated to achieve targeted release profiles [11]. The choice of polymeric carrier plays a significant role in regulating release behavior since physicochemical properties such as hydrophobicity, crystallinity, and functional groups will determine drug–polymer interaction.

For the purpose of the research in this paper, we decided to use the polymers mentioned initially (PLA and PCL), and chitosan, which have good reported biocompatibility and FDA-approved clinical use. Chitosan is a naturally occurring polysaccharide with built-in antimicrobial properties and potential for functional derivative modification and synergistic action [13,14]. The blending of these polymers together in hybrid systems provides a greater degree of freedom so that one can design degradation kinetics and drug release profiles. These concepts have already shown potential in drug delivery systems, yet their application in vascular and cardiovascular surgery sutures has not been fully explored. The project builds upon these concepts by developing a bioactive, biodegradable polymeric coating for surgical sutures designed for vascular and cardiovascular applications. The project will enable local and controlled delivery of active antiproliferative and immunosuppressive drugs, including tacrolimus, sirolimus, and paclitaxel [15,16]. These drugs were selected due to their fully characterized potential to inhibit neointimal hyperplasia, vascular restenosis, and inflammation—the pathologies that are central clinical problems following vascular interventions. These compounds differ in their physicochemical properties and mechanisms of action, allowing assessment of how drug characteristics influence incorporation and release behavior from various polymer matrices. Moreover, all three agents are clinically relevant in vascular applications due to their antiproliferative and anti-inflammatory effects. By investigating PLA, PCL, and chitosan in a systematic fashion, both as neat single-component polymers and in binary mixtures, the project has the goal of drawing correlations between drug release behavior and polymer physico-chemical properties. Through this approach, the project addresses the fundamental question of how therapeutic performance is influenced by the choice of polymers and formulation, thereby driving the rational design of future drug-eluting sutures. Eventually, this approach has the capability to improve surgical outcomes, reduce the incidence of post-operative complications, and expand the utility of absorbable sutures in cardiovascular medicine. To the best of our knowledge, this study provides a systematic comparison of multiple clinically relevant drugs across a wide range of biodegradable polymer compositions, enabling evaluation of both formulation- and drug-dependent release behavior.

## 2. Results

### 2.1. Characterization of Polymeric Coatings and Drug Loading

Polymeric coatings incorporating sirolimus, tacrolimus, or paclitaxel were successfully deposited onto PLA-based surgical sutures using a solvent-mediated immersion and drying procedure. The coating process resulted in continuous coatings with no visible macroscopic defects such as cracking or delamination. A total of 36 coated suture variants were prepared, corresponding to twelve polymer compositions for each drug, including single-polymer systems (PLA, PCL, and chitosan) and their binary blends, as summarized in Table 1. All release experiments were performed in triplicate (*n* = 3), and results are presented as mean values.

The applied coating method enabled reproducible incorporation of all three drugs into biodegradable polymer matrices. The use of different polymer compositions and blend ratios allowed systematic variation in coating formulation while maintaining consistent preparation conditions. All polymer systems formed stable coatings on the suture surface and remained intact during handling and sample preparation, confirming adequate coating adhesion and structural integrity.

Differences in polymer composition were expected to influence drug distribution within the coating matrix due to variations in physicochemical properties such as hydrophobicity, polymer compatibility, and matrix structure. Single-polymer coatings provided homogeneous polymer environments, while binary blends enabled the formation of mixed polymer matrices with potentially distinct drug–polymer interactions. These formulation differences provided a basis for further evaluation of their influence on drug release behavior.

Collectively, these results confirm successful and reproducible preparation of drug-loaded polymer-coated sutures using biodegradable polymers with diverse physicochemical properties, supporting their suitability for controlled drug delivery applications.

### 2.2. In Vitro Drug Release Kinetics

The in vitro drug release profiles demonstrated sustained and formulation-dependent release of sirolimus, tacrolimus, and paclitaxel over the 12-week observation period. All formulations exhibited progressive cumulative release without evidence of premature depletion, confirming the capacity of the polymer coatings to provide extended drug delivery. All release experiments were performed in triplicate (n = 3), and results are presented as mean values.

#### 2.2.1. Release Kinetics of Sirolimus-Loaded Sutures

**Figure 1:**
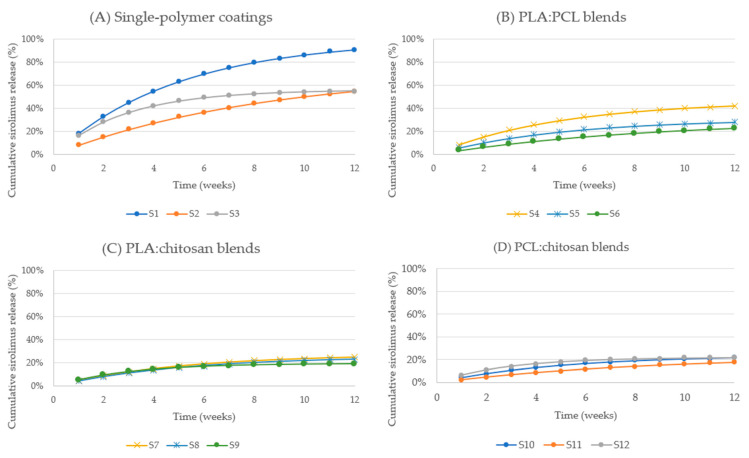
Cumulative in vitro release of sirolimus from polymer-coated surgical sutures over 12 weeks: (**A**) single polymers, (**B**) PLA:PCL blends, (**C**) PLA:chitosan blends, and (**D**) PCL:chitosan blends.

Sirolimus release profiles exhibited distinct initial faster release phase followed by sustained release (Figure 1). The magnitude of the initial phase varied substantially depending on polymer composition. Pure PLA coatings (S1) demonstrated rapid early release, reaching 18.13% (2.05 µg) within the first week and 32.97% (3.73 µg) by week 2. This was followed by continued sustained release, reaching 55.07% by week 4, 75.34% by week 7, and ultimately 90.93% (10.28 µg) by week 12.

In contrast, PCL-based coatings (S2) exhibited significantly slower release kinetics, with cumulative release of only 8.08% (0.81 µg) by week 1 and 27.23% (2.72 µg) by week 4. Sustained release continued through week 12, reaching 54.51% (5.45 µg). Similarly, PCL-rich binary blends such as PLA:PCL 1:2 (S6) exhibited markedly reduced cumulative release, reaching only 22.60% (2.26 µg) by week 12.

Chitosan-based coatings (S3) demonstrated intermediate release kinetics, with cumulative release reaching 16.52% by week 1, 42.15% by week 4, and 55.11% (5.51 µg) by week 12. Binary blends containing chitosan generally exhibited lower cumulative release compared to pure PLA formulations, particularly PCL:chitosan blends, which demonstrated cumulative release values below 20% in certain formulations.

Across all sirolimus-loaded formulations, cumulative release continued throughout the entire study duration, with no plateau observed prior to week 12, indicating sustained drug availability over prolonged periods.

#### 2.2.2. Release Kinetics of Tacrolimus-Loaded Sutures

**Figure 2:**
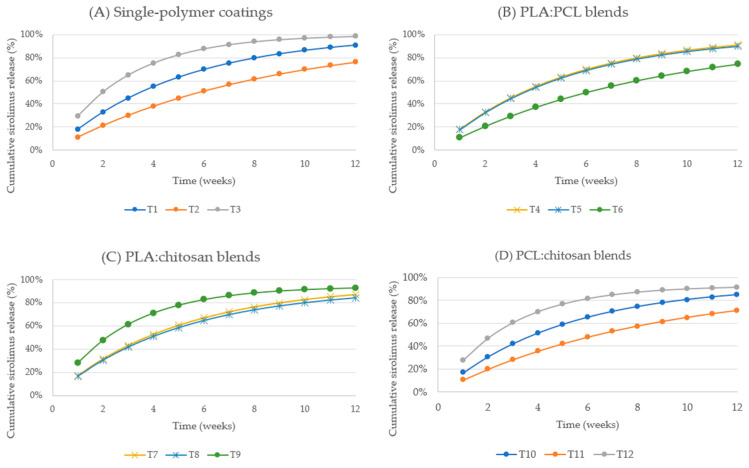
Cumulative in vitro release of tacrolimus from polymer-coated surgical sutures over 12 weeks: (**A**) single polymers, (**B**) PLA:PCL blends, (**C**) PLA:chitosan blends, and (**D**) PCL:chitosan blends.

Tacrolimus-loaded formulations demonstrated faster release kinetics compared to sirolimus, with several polymer compositions approaching near-complete drug release (Figure 2). Pure chitosan coatings (T3) exhibited the most rapid release, reaching 29.53% (3.13 µg) within the first week and exceeding 50.34% (5.34 µg) by week 2. Continued release resulted in cumulative values of 75.34% by week 4, 91.37% by week 7, and 98.50% (10.45 µg) by week 12.

Pure PLA coatings (T1) also demonstrated rapid release, reaching 18.13% by week 1, 55.07% by week 4, and 90.93% (10.17 µg) by week 12. PCL-based coatings (T2) exhibited slower initial release, reaching 11.31% by week 1 and 38.12% by week 4, but continued sustained release resulted in cumulative release of 76.31% (8.37 µg) by week 12.

Binary polymer blends demonstrated tunable release kinetics. PLA:PCL blends exhibited moderate release rates, while chitosan-containing blends such as PLA:chitosan (T7, T8) exhibited sustained release reaching 86.96% and 84.36%, respectively, by week 12. PCL:chitosan blends demonstrated comparatively slower release, although cumulative release still exceeded 70% across all formulations.

Tacrolimus release profiles showed continuous cumulative increases over the entire observation period, with most formulations maintaining measurable release beyond week 8, indicating prolonged release capability.

#### 2.2.3. Release Kinetics of Paclitaxel-Loaded Sutures

**Figure 3:**
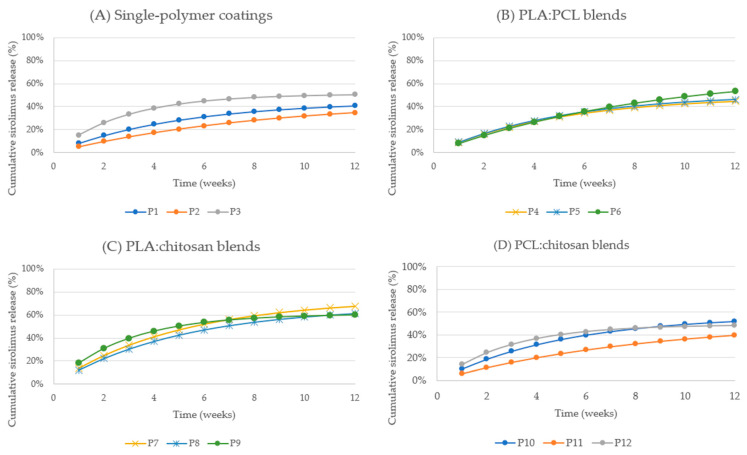
Cumulative in vitro release of paclitaxel from polymer-coated surgical sutures over 12 weeks: (**A**) single polymers, (**B**) PLA:PCL blends, (**C**) PLA:chitosan blends, and (**D**) PCL:chitosan blends.

Paclitaxel release profiles demonstrated slower release kinetics compared to sirolimus and tacrolimus across all polymer compositions (Figure 3). Pure PLA coatings (P1) reached cumulative release of 8.14% (0.814 µg) by week 1, increasing gradually to 24.72% by week 4 and 40.82% (4.08 µg) by week 12.

PCL-based coatings (P2) exhibited slower release, reaching 5.13% by week 1, 17.30% by week 4, and 34.62% (3.46 µg) by week 12. In contrast, chitosan-containing blends demonstrated enhanced cumulative release. PLA:chitosan 1:1 coatings (P7) exhibited the highest cumulative release, reaching 13.49% by week 1, 40.97% by week 4, and 67.65% (6.77 µg) by week 12.

Similarly, PLA:chitosan 2:1 and PLA:chitosan 1:2 blends (P8, P9) achieved cumulative release exceeding 60% by week 12. PCL:chitosan blends exhibited intermediate performance, with cumulative release ranging from 39.80% to 48.20%.

Paclitaxel release profiles demonstrated sustained cumulative release throughout the entire study period, without early plateau formation, confirming prolonged drug release capability.

#### 2.2.4. Comparative Analysis of Release Kinetics Across Drugs and Polymer Compositions

Comparison across drug types revealed clear differences in release kinetics and cumulative release capacity. As shown in Table 2, tacrolimus exhibited the highest cumulative release, reaching up to 98.50%, followed by sirolimus, which reached up to 90.93%, and paclitaxel, which reached up to 67.65%. These differences were consistent across polymer compositions and timepoints. Polymer composition exerted a strong influence on release kinetics across all drugs. PLA-based coatings generally demonstrated faster release, while PCL-based coatings exhibited slower, more sustained release. Chitosan-containing formulations exhibited variable release kinetics depending on blend composition, with several formulations demonstrating enhanced cumulative release compared with single-polymer systems. Across all drugs and formulations, cumulative release progressed continuously throughout the 12-week study period, confirming the ability of the polymer coatings to support prolonged drug release under in vitro conditions. To further elucidate the drug release mechanism, cumulative release data were fitted to the Higuchi and Korsmeyer–Peppas models for all formulations. Selected representative results are presented in Table 2, while the complete dataset is provided in Supplementary Table S1. In general, the Higuchi model showed good to excellent agreement with the experimental data, indicating a strong contribution of diffusion-controlled release. Korsmeyer–Peppas analysis yielded release exponent (n) values consistent with predominantly diffusion-controlled or anomalous transport, depending on formulation composition. The obtained n values (Table 2) suggest predominantly Fickian or quasi-Fickian diffusion behavior.

### 2.3. Effect of Polymer Composition on Release Profile

Polymer composition exerted a pronounced influence on both the magnitude and temporal progression of drug release across all three active compounds. Distinct release behaviors were observed for single-component polymers (PLA, PCL, chitosan) and their binary blends, demonstrating the ability to modulate drug release kinetics through rational selection of polymer matrices.

Clear differences in release performance were observed among single-polymer systems. PLA-based coatings consistently demonstrated higher cumulative release compared with PCL-based coatings for all tested drugs (Table 3). For sirolimus, pure PLA coatings (S1) achieved cumulative release of 90.93%, whereas pure PCL coatings (S2) reached only 54.51% by week 12. Similar trends were observed for tacrolimus, with PLA coatings (T1) achieving 90.93% cumulative release compared to 76.31% for PCL coatings (T2). Paclitaxel release also followed this trend, with PLA-based coatings (P1) reaching 40.82%, while PCL coatings (P2) achieved only 34.62% cumulative release by week 12. These findings demonstrate that PCL-based coatings provided stronger drug retention compared with PLA-based coatings. Chitosan-based coatings exhibited compound-dependent release behavior. For tacrolimus, chitosan coatings (T3) demonstrated the highest cumulative release (98.50%), whereas for sirolimus and paclitaxel, cumulative release from chitosan coatings reached 55.11% (S3) and 50.44% (P3), respectively, indicating efficient but compound-specific release performance.

Binary blends of PLA and PCL demonstrated intermediate release behavior between the respective single-polymer systems, confirming tunable release performance through polymer blending. For sirolimus, cumulative release from PLA:PCL blends ranged from 22.60% (PLA:PCL 1:2; S6) to 42.01% (PLA:PCL 1:1; S4) and 28.03% (PLA:PCL 2:1; S5) by week 12. These results indicate that increasing PCL content reduced cumulative release, while balanced PLA:PCL compositions allowed moderate sustained release. Tacrolimus-loaded PLA:PCL blends demonstrated cumulative release ranging from 74.47% (PLA:PCL 1:2; T6) to 90.93% (PLA:PCL 1:1; T4), confirming efficient drug release while maintaining sustained release characteristics. Similar trends were observed for paclitaxel, with cumulative release values of 53.19% (PLA:PCL 1:2; P6), 44.73% (PLA:PCL 1:1; P4), and 46.10% (PLA:PCL 2:1; P5). These findings demonstrate that increasing the proportion of PCL within PLA:PCL blends resulted in slower release kinetics and reduced cumulative release.

Blends containing chitosan demonstrated distinct release modulation effects depending on the co-polymer composition. PLA:chitosan blends exhibited enhanced release compared with PCL:chitosan blends across all drugs. For paclitaxel, PLA:chitosan 1:1 (P7) achieved the highest cumulative release among all paclitaxel formulations (67.65%), followed by PLA:chitosan 2:1 (P8) and PLA:chitosan 1:2 (P9), which achieved cumulative release exceeding 60%. Similarly, tacrolimus-loaded PLA:chitosan blends demonstrated cumulative release values of 86.96% (T7) and 84.36% (T8), confirming efficient drug release from these matrices. In contrast, PCL:chitosan blends demonstrated lower cumulative release across all drugs. For example, sirolimus release from PCL:chitosan blends ranged from 17.53% (S11) to 23.20% (S8), representing the lowest cumulative release observed among all polymer systems tested. Paclitaxel-loaded PCL:chitosan blends demonstrated cumulative release ranging from 39.80% (P11) to 48.20% (P12), while tacrolimus release ranged from 70.93% (T11) to 91.56% (T12), confirming compound-dependent release modulation by chitosan-containing blends.

Overall, polymer composition enabled precise modulation of cumulative drug release across all tested compounds. PLA-based coatings generally supported higher cumulative release, while PCL-containing coatings provided slower release and enhanced drug retention. Binary blends allowed intermediate release profiles, and chitosan-containing blends provided additional flexibility in tuning release performance.

These results demonstrate that polymer selection and composition represent critical determinants of drug release kinetics, enabling controlled modulation of release behavior over extended periods.

### 2.4. Mechanical Integrity and Coating Stability

Mechanical properties and coating stability of the sutures before and after coating were qualitatively assessed through handling, ex vivo suturing tests, and visual inspection after immersion and mechanical manipulation. All tested formulations formed continuous coatings with no visible macroscopic defects on the suture surface, such as cracking, delamination, or discontinuities immediately following coating and drying procedures. The coated sutures exhibited comparable handling characteristics to uncoated sutures, with no noticeable loss of flexibility or increased stiffness during manipulation.

During ex vivo suturing tests, coated sutures were passed through biological tissue to assess coating adhesion and structural integrity under conditions simulating clinical use. Formulations containing PLA as a major polymer component demonstrated excellent coating stability, maintaining continuous and intact coatings throughout the suturing procedure without visible signs of coating disruption or material transfer to the tissue surface.

In contrast, coatings containing higher proportions of PCL exhibited reduced adhesion to the suture substrate. In these formulations, localized coating deformation and partial surface irregularities were occasionally observed following mechanical manipulation, although coating continuity remained preserved in most cases. These observations suggest that polymer composition influenced coating adhesion and mechanical robustness.

Chitosan-containing formulations demonstrated acceptable mechanical stability, although their performance varied depending on blend composition. PLA-containing blends generally exhibited superior coating integrity compared to PCL-dominated blends, consistent with differences in polymer mechanical properties and adhesion characteristics.

Importantly, the optimized formulations selected for further development demonstrated stable coating morphology and maintained coating integrity throughout mechanical handling and suturing procedures. No complete coating detachment or catastrophic coating failure was observed in any of the tested formulations.

These findings confirm that the applied coating process produced mechanically stable drug-loaded polymer coatings capable of withstanding handling and simulated clinical manipulation, supporting their suitability for further development as drug-eluting surgical sutures. These observations indirectly support coating continuity and adhesion under conditions relevant to practical use. However, these observations should be considered preliminary, and quantitative mechanical characterization (e.g., tensile strength or flexibility testing) will be required in future studies.

## 3. Discussion

The present study demonstrates that biodegradable polymer coatings based on PLA, PCL, chitosan, and their binary blends enable controlled and sustained release of sirolimus, tacrolimus, and paclitaxel from surgical sutures over an extended period of up to 12 weeks. The use of multiple drugs further highlights the versatility of the proposed polymer coating system as a platform for tunable drug delivery. The results confirm that polymer composition plays a critical role in modulating drug release kinetics, cumulative release, and release duration, providing a versatile platform for tunable local drug delivery. A limitation of the present study is the lack of dedicated degradation studies for the investigated coating systems. Although the release profiles were analyzed over 12 weeks and interpreted in the context of known polymer properties, direct evaluation of degradation kinetics will be necessary in future studies to better distinguish between diffusion-controlled and degradation-assisted release mechanisms.

A key finding of this study is the pronounced effect of polymer composition on release kinetics across all tested drugs. Pure PLA coatings generally exhibited faster and more extensive drug release compared to PCL-based systems. This behavior may be related to the differences in physicochemical properties of the polymers, including differences in their reported degradation rate and hydrophilicity, particularly between PLA and PCL, which are known to affect water penetration and drug diffusion in polymer matrices. Because degradation studies were not performed in the present work, the contribution of polymer erosion to the observed release profiles should be interpreted with caution. Therefore, the proposed release mechanisms should be considered as indicative rather than definitive and require further experimental validation. In contrast, PCL-containing formulations consistently demonstrated slower release kinetics and lower cumulative release, reflecting the more hydrophobic nature and slower degradation rate of PCL. Similar observations have been reported in previous studies, where increasing PCL content reduced drug release rate due to slower polymer degradation and reduced diffusion pathways.

The interactions between drugs and polymer matrices are likely governed by physicochemical properties such as hydrophobicity and molecular affinity, which may influence drug retention and diffusion within the polymer network. The stability of the incorporated drugs within the polymeric matrices and their potential interactions with the polymer components were not directly evaluated in the present study. However, the sustained and reproducible release profiles observed over the 12-week period suggest that the active compounds remained stable within the coating matrix under the applied experimental conditions. The absence of abrupt release drops or irregular release patterns suggests that no significant drug degradation occurred during the release study.

Sirolimus, tacrolimus, and paclitaxel are highly hydrophobic compounds, which may promote their affinity toward hydrophobic polymer domains, particularly in PLA- and PCL-based systems. Such interactions can contribute to reduced diffusion rates and prolonged drug retention within the matrix. In contrast, the presence of chitosan, a more hydrophilic and functionalized polymer, may alter drug–polymer interactions and facilitate increased drug mobility and release.

Nevertheless, detailed investigation of drug stability and molecular-level interactions (e.g., using spectroscopic or thermal analysis techniques) is required to confirm these hypotheses and will be the subject of future studies.

Chitosan-containing formulations exhibited distinct release behavior, particularly for tacrolimus and paclitaxel, where several chitosan-based blends demonstrated enhanced cumulative release compared with single-polymer systems. This effect may be associated with the hydrophilic and porous structure of chitosan, which facilitates water penetration and drug diffusion through the polymer matrix. Additionally, chitosan is known to exhibit favorable swelling properties, which can increase drug mobility and enhance sustained release performance.

All tested formulations exhibited sustained release profiles characterized by an initial faster release phase followed by prolonged release over the remaining study period. The initial faster release phase, observed during the first week, likely corresponds to diffusion of drug molecules located near or at the coating surface. This behavior is frequently observed in polymer-based drug delivery systems and is primarily governed by drug distribution within the polymer matrix and surface accessibility.

Following the initial phase, drug release proceeded in a sustained and progressive manner over the full 12-week period. This sustained release phase is primarily governed by a combination of drug diffusion through the polymer matrix and gradual polymer degradation. Previous studies have demonstrated that drug release from biodegradable polymer coatings is typically controlled by a combination of diffusion-controlled and degradation-controlled mechanisms.

The relative contribution of these mechanisms depends on polymer composition, crystallinity, hydrophobicity, and degradation kinetics. Comparison between the three tested drugs revealed clear compound-dependent differences in release behavior. Tacrolimus exhibited the highest cumulative release across most formulations, reaching up to 98.50% cumulative release, while sirolimus demonstrated intermediate release, and paclitaxel exhibited the lowest cumulative release. These differences may be attributed to variations in physicochemical properties, including molecular weight, hydrophobicity, and drug–polymer interactions, which influence diffusion rates and polymer affinity. Paclitaxel, being highly hydrophobic, likely exhibits stronger interactions with hydrophobic polymer domains, resulting in slower release kinetics and enhanced retention within the polymer matrix. These interpretations are based on observed release trends and literature data and should be considered indicative rather than definitive, as no direct experimental measurements of degradation kinetics or diffusion parameters were performed.

Binary polymer blends demonstrated particularly effective modulation of drug release kinetics, confirming the ability to fine-tune release performance by adjusting polymer composition. PLA:PCL blends provided intermediate release profiles between the faster-releasing PLA systems and slower-releasing PCL systems. This behavior is consistent with previous reports demonstrating that blending polymers with different degradation rates provides an effective strategy for controlling drug release duration and kinetics.

Importantly, sustained drug release was observed throughout the entire 12-week study period, with no evidence of premature release plateau or complete drug depletion in most formulations. This prolonged release profile is highly relevant for clinical applications involving surgical sutures, where extended local drug delivery may help prevent restenosis, inflammation, and excessive tissue proliferation following surgical intervention.

Mechanical stability observations further confirmed that optimized polymer formulations maintained coating integrity during handling and suturing procedures, supporting their suitability for practical surgical applications. Stable coating integrity is essential to ensure consistent drug delivery performance and prevent premature coating failure. Nevertheless, a limitation of the present study is the lack of detailed morphological characterization of the coatings (e.g., SEM analysis), which would provide quantitative information on coating thickness, surface structure, and uniformity. An additional limitation of the present study is the lack of dedicated biodegradation and biocompatibility studies of the developed coating systems. Although the polymers used (PLA, PCL, and chitosan) are well-known for their biocompatibility and biodegradability, further in vitro and in vivo studies will be necessary to confirm the biological safety and performance of the proposed drug-eluting sutures. However, further studies addressing biodegradation and biocompatibility will be required prior to clinical translation.

Collectively, these findings demonstrate that polymer composition provides a powerful tool for controlling drug release from coated surgical sutures. By selecting appropriate polymer systems and blend ratios, it is possible to achieve a broad range of release profiles tailored to specific clinical requirements. This tunable drug delivery capability highlights the potential of polymer-coated sutures as a versatile platform for localized, sustained drug delivery in surgical and vascular applications.

The investigated drugs—sirolimus, tacrolimus, and paclitaxel—are widely used in cardiovascular applications, particularly in drug-eluting stents, where they play a key role in reducing restenosis through antiproliferative and anti-inflammatory effects. In the present study, the obtained sustained release profiles suggest the potential applicability of the developed system for localized drug delivery in vascular interventions. However, the study does not address therapeutic concentration ranges, direct comparison with existing drug-eluting systems, or potential local toxicity. These aspects will require further in vitro and in vivo investigations to fully evaluate the clinical relevance of the proposed approach.

## 4. Materials and Methods

### 4.1. Instrumentation

Formic acid and ammonium formate were purchased from Sigma-Aldrich (St. Louis, MO, USA). Acetonitrile (LC-MS grade, ≥99.9% purity) was obtained from Honeywell (Morris Plains, NJ, USA). Ultrapurified water, with a resistivity of 18.2 MΩ·cm, was generated using a Milli-Q Water Purification System (Merck Millipore, Burlington, MA, USA).

Polymeric excipients included poly(L-lactic acid) (PLA; average Mw ~75,000; Sigma-Aldrich, Darmstadt, Germany, Cat. No. 38534), polycaprolactone (PCL; average Mw ~80,000; Sigma-Aldrich, Darmstadt, Germany, Cat. No. 704105), and chitosan (medium molecular weight; Sigma-Aldrich, Darmstadt, Germany, Cat. No. 448877). All polymers were used as received without further purification.

The active pharmaceutical ingredients—sirolimus, tacrolimus, and paclitaxel—were purchased from Merck (Sigma-Aldrich, St. Louis, MO, USA) at analytical grade. Tween^®^ 80 (polysorbate 80; Merck, Sigma-Aldrich, Darmstadt, Germany; Cat. No. 817061) was used as a surfactant for solubilization of active compounds prior to incorporation into polymer coating solutions. Dichloromethane (≥99.8% purity; Merck, Sigma-Aldrich, Darmstadt, Germany; Cat. No. 106044) was employed as an organic solvent for dissolution of PLA and PCL.

Phosphate-buffered saline (PBS; pH 7.4; Sigma-Aldrich, Darmstadt, Germany, Cat. No. P4474) was used as the release medium during in vitro drug release studies. All chemicals and reagents were of analytical or higher purity and were used as received.

Sample preparation and chromatographic analysis were performed using a high-performance liquid chromatography (HPLC) system (Shimadzu, Kyoto, Japan) equipped with dual high-precision solvent feed pumps with a gradient capability (LC-30AD), an in-line degasser (DGU-20A5R) to remove dissolved gases from the mobile phase, an autosampler (SIL-30AC) enabling accurate and reproducible sample injection, a thermostated column oven (CTO-20AC) for maintaining constant separation temperature, and a UV-VIS detector (SPD-20AD) for quantitative analyte determination. Separations were carried out on a reversed-phase Kinetex C18 column (150 × 4.6 mm; Phenomenex, Torrance, CA, USA) characterized by core–shell particle technology, ensuring high efficiency and reduced backpressure.

Ultrapure water of 18.2 MΩ·cm resistivity was produced using a Milli-Q Water Purification System (Merck Millipore, Burlington, MA, USA) and used in the preparation of mobile phases and sample dilutions. Gravimetric measurements were conducted on an analytical balance (XA 82/220.R2, Radwag, Radom, Poland) with a readability of 0.01 mg, ensuring high weighing accuracy. For sample agitation and incubation under controlled conditions, an orbital platform shaker (Unimax 1010, Heidolph, Schwabach, Germany) was employed. Homogenization of polymer coating formulations was carried out using a high-performance vortex mixer (Multi Reax, Heidolph, Schwabach, Germany), enabling reproducible dispersion of active pharmaceutical ingredients within the polymer matrix.

Formulations containing cytostatic or immunosuppressive agents were prepared under controlled environmental and sterility conditions in a Class II biological safety cabinet (SafeMate Eco+, BioAir S.p.A., Siziano, PV, Italy), ensuring operator protection and prevention of cross-contamination. Surgical sutures coated with polymer formulations were dried in a precision laboratory drying oven (FD series, model FD 115, Binder GmbH, Tuttlingen, Germany) equipped with forced-air convection to guarantee uniform temperature distribution. All glassware used during the experiments was subjected to overnight drying in a temperature-controlled oven, followed by cooling with a high-purity nitrogen stream to eliminate moisture prior to use.

Pipetting procedures were performed using adjustable-volume micropipettes (Tacta, Sartorius Biohit, Göttingen, Germany) for precise liquid handling, complemented by an electronic dispensing system (Multipette E3, Eppendorf, Hamburg, Germany) to ensure reproducibility in the preparation of calibration standards, analytical samples, and coating solutions.

### 4.2. Preparation of Drug-Loaded Polymeric Coatings on PLA-Based Surgical Sutures

In this study, bioactive polymeric coatings were developed and applied onto commercially available surgical sutures composed of poly(L-lactic acid) (PLA). Three polymeric carriers—poly(L-lactic acid) (PLA), polycaprolactone (PCL), and chitosan—were selected on the basis of their proven biocompatibility, tunable degradation kinetics, and established use in biomedical applications. The selection of these polymers allowed for systematic evaluation of the influence of hydrophobicity, crystallinity, and chemical functionality on drug incorporation efficiency and release kinetics.

A total of twelve distinct coating formulations were prepared, encompassing single-component systems based on PLA, PCL, or chitosan, as well as binary polymeric blends of PLA + PCL, PLA + chitosan, and PCL + chitosan in weight ratios of 1:1, 1:2, and 2:1. This comprehensive formulation matrix enabled a systematic evaluation of the individual physicochemical and functional properties of each polymer, while also allowing the investigation of potential synergistic effects arising from their combination in mixed systems.

For the preparation of coating solutions, each polymer (or polymer blend) was weighed with analytical precision (±0.01 mg) and dissolved in an appropriate solvent system. PLA and PCL were solubilized in dichloromethane, whereas chitosan was dissolved in an aqueous 1% (*w*/*v*) formic acid solution to protonate amino groups and facilitate dissolution. For binary systems, polymer quantities were proportionally adjusted to achieve the intended weight ratio, and solvents were combined in corresponding volumetric proportions to ensure complete dissolution of both constituents. The target polymer concentration in each coating solution was maintained at 20 mg/mL.

Three pharmacologically active agents—sirolimus, tacrolimus, and paclitaxel—were incorporated into the polymeric coatings. These drugs were selected for their potent antiproliferative, immunosuppressive, and antineoplastic activities, relevant to the prevention of post-surgical restenosis, neointimal hyperplasia, and inflammatory responses. Each drug (20 mg) was first dissolved in polysorbate 80 (Tween^®^ 80) to form a homogenous, surfactant-based drug concentrate, yielding a final concentration of approximately 1.95% (*w*/*w*). The drug–Tween^®^ 80 concentrates were subsequently added to the polymer solutions and mixed thoroughly to ensure uniform molecular dispersion of the active substance within the coating matrix.

Prior to coating deposition, the PLA-based surgical sutures were cut into 1 cm segments and subjected to a pre-treatment process designed to optimize coating adhesion. Sutures were immersed in ethanol to remove residual surface oils, lubricants, and potential manufacturing contaminants. Following this degreasing step, the sutures were dried at 60 °C for 30 min in a forced-air drying oven, a procedure intended to remove residual solvent and induce slight surface micro-roughening, thereby enhancing polymer adhesion.

The coating process was performed by immersing the pretreated suture segments into the respective drug-loaded polymer solutions for 24 h under controlled environmental conditions to allow for complete adsorption and entrapment of the coating material. After immersion, the coated sutures were carefully removed from the coating medium, subjected to gentle mechanical agitation to prevent aggregation, and placed in a controlled environment to facilitate solvent evaporation without compromising coating integrity.

**Figure 4:**
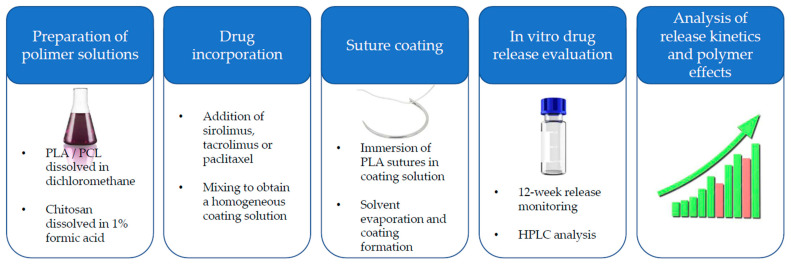
Schematic illustration of the preparation and evaluation of drug-eluting polymer-coated surgical sutures, including polymer solution preparation, drug incorporation, suture coating, and subsequent in vitro drug release analysis.

This methodology yielded a total of thirty-six distinct bioactive-coated suture variants, representing the full factorial combination of twelve polymer compositions and three incorporated APIs. These prepared specimens were subsequently subjected to subsequent evaluation of drug release behavior and functional performance to elucidate the influence of polymer composition and drug type on coating properties, adhesion, mechanical performance, and drug release kinetics (Figure 4).

### 4.3. Chromatographic Analysis

Quantitative analyses of drug content in coated PLA-based surgical sutures were performed using high-performance liquid chromatography (HPLC). All measurements were conducted on a Shimadzu liquid chromatograph equipped with a UV/VIS detector, autosampler, and thermostatted column compartment.

Chromatographic separation was carried out under isocratic elution conditions. The mobile phase consisted of 25% (*v*/*v*) aqueous 10 mM ammonium formate containing 0.1% (*v*/*v*) formic acid (solvent A) and 75% (*v*/*v*) acetonitrile containing 0.1% (*v*/*v*) formic acid (solvent B). The flow rate was set to 1.0 mL/min, and the column temperature was maintained at 35 °C. An injection volume of 30 µL was used for all samples. Detection was performed at 230 nm.

Under these chromatographic conditions, the retention times for the investigated active pharmaceutical ingredients were approximately 2.53 min for tacrolimus, 4.64 min for sirolimus, and 3.59 min for paclitaxel. Prior to analysis, the system was equilibrated for a minimum of 20 min under the specified mobile phase composition. Following each analytical series, the system was flushed sequentially with acetonitrile and ultrapure water to remove residual analytes and prepare the column for subsequent runs or shutdown.

All chromatographic procedures were internally validated in accordance with the ICH Q2(R1) guidelines. The validation parameters included linearity (R^2^ > 0.999), accuracy (recoveries within 98–102%), precision (RSD < 2%), limit of detection (LOD), and limit of quantification (LOQ), all of which met the predefined acceptance criteria.

### 4.4. In Vitro Drug Release Studies

The in vitro release kinetics of sirolimus, tacrolimus, and paclitaxel from bioactive polymer-coated PLA-based surgical sutures were investigated under controlled dynamic conditions over a 12-week period. Each experiment was performed in triplicate (*n* = 3). Release experiments were conducted in phosphate-buffered saline (PBS, pH 7.40 ± 0.05; Sigma-Aldrich, analytical grade), selected to simulate physiological ionic strength and pH.

Prior to initiation of the release study, each coated suture specimen was accurately weighed and placed individually into sterile, chemically inert borosilicate glass vials (2 mL nominal capacity), previously rinsed with ultrapure water and dried under a nitrogen stream to minimize any potential adsorption of analytes to the container surface. Each vial was filled with 2.0 mL of pre-warmed PBS, ensuring complete immersion of the suture segment without mechanical tension or coiling that could alter the exposed surface area.

Incubation was carried out in a temperature-controlled orbital shaker (Unimax 1010, Heidolph, Schwabach, Germany) maintained at 37.0 ± 0.5 °C with a rotation speed of 100 rpm. This setting was selected to provide uniform hydrodynamic conditions around the suture surface while avoiding excessive shear stress that could mechanically disrupt the coating. The shaker platform was enclosed to minimize evaporative losses and to maintain constant temperature throughout the experiment.

Sampling was performed at predetermined time intervals of 7 days, up to a total duration of 12 weeks. At each time point, the entire release medium was carefully withdrawn using a calibrated adjustable-volume micropipette, ensuring the avoidance of bubble formation or contact of the pipette tip with the suture surface, which could lead to loss of coating material. The withdrawn medium was transferred into amber autosampler vials to protect the analytes from light degradation and stored at 4 °C until chromatographic analysis, which was conducted within 24 h of collection. Immediately after sampling, the release medium in each vial was replaced with an identical volume of fresh, pre-warmed PBS to maintain sink conditions and constant ionic strength.

Quantitative analysis of the released drug was performed using a validated high-performance liquid chromatography (HPLC) protocol described above. For each analyte, calibration curves were constructed over the relevant concentration ranges using freshly prepared standards in PBS. All experiments were performed in triplicate for each suture–drug combination (n = 3), and results were expressed as both the cumulative amount released (µg) and the corresponding percentage of the total drug load initially present in the coated suture.

To analyze the release mechanism, cumulative release data were fitted to the Higuchi and Korsmeyer–Peppas models. Higuchi fitting was performed by plotting the cumulative fractional release against the square root of time using the complete release profile. Korsmeyer–Peppas fitting was performed using the log-transformed equation for the initial release phase, considering only data points with Mt/M∞ ≤ 0.6. Linear regression was used to determine model parameters and coefficients of determination (R^2^).

## 5. Conclusions

This study demonstrates the feasibility of developing biodegradable polymer-coated surgical sutures capable of providing controlled and sustained release of therapeutically relevant agents, including sirolimus, tacrolimus, and paclitaxel. The applied solvent-based coating approach enabled efficient incorporation of all tested drugs into polymer matrices composed of PLA, PCL, chitosan, and their binary blends, resulting in reproducible and formulation-dependent drug release profiles over a prolonged period of up to 12 weeks.

The results clearly demonstrate that polymer composition plays a key role in regulating drug release kinetics and cumulative release. PLA-based coatings generally exhibited faster release profiles, while PCL-containing formulations provided slower and more sustained release, reflecting differences in polymer physicochemical properties such as hydrophobicity and degradation rate. Binary polymer blends enabled precise modulation of release behavior, confirming that polymer composition can be used as an effective tool to tailor drug delivery performance to specific therapeutic requirements. Chitosan-containing formulations further expanded the range of achievable release profiles, demonstrating the versatility of the polymer platform.

Importantly, the developed coatings maintained structural integrity during handling and simulated suturing procedures, indicating their suitability for practical surgical applications. The sustained release observed across all tested formulations suggests that such systems may provide prolonged local drug exposure, which is particularly relevant for preventing restenosis, excessive tissue proliferation, and inflammatory responses following vascular and surgical interventions.

Overall, these findings establish biodegradable polymer-coated sutures as a promising and tunable drug delivery platform. The ability to control drug release through rational polymer selection provides a strong foundation for further optimization and translational development. Future studies should focus on in vivo evaluation, long-term biocompatibility, and therapeutic efficacy to support the clinical translation of drug-eluting sutures for improved surgical outcomes.

## 6. Patents

The work reported in this manuscript is related to a patent application entitled “Nić chirurgiczna powleczona kompozycją polimerową” (English: “Surgical suture coated with a polymer composition”), filed by Medesto Sp. z o.o. with the Polish Patent Office (Urząd Patentowy Rzeczypospolitej Polskiej). The patent application was filed on 30 September 2025 and assigned application number P.453380. The invention relates to polymer-coated surgical sutures designed for controlled and sustained drug delivery.

## Figures and Tables

**Table 1:** Composition of polymer coating formulations used for preparation of drug-eluting surgical sutures.

Formulation No	Polymer Composition	Polymer Ratio
1	PLA	n/a
2	PCL	n/a
3	Chitosan	n/a
4	PLA + PCL	1:1
5	PLA + PCL	2:1
6	PLA + PCL	1:2
7	PLA + Chitosan	1:1
8	PLA + Chitosan	2:1
9	PLA + Chitosan	1:2
10	PCL + Chitosan	1:1
11	PCL + Chitosan	2:1
12	PCL + Chitosan	1:2

n/a: not applicable.

**Table 2:** Kinetic modeling parameters for selected representative formulations.

Formulation	Drug	Higuchi kH	Higuchi R^2^	Peppas n	Peppas R^2^
S1	Sirolimus	0.299	0.981	0.806	0.998
S2	Sirolimus	0.194	0.998	0.761	0.991
S3	Sirolimus	0.149	0.906	0.461	0.927
T1	Tacrolimus	0.299	0.981	0.806	0.998
T2	Tacrolimus	0.271	0.998	0.831	0.997
P1	Paclitaxel	0.134	0.981	0.636	0.974
P2	Paclitaxel	0.123	0.998	0.761	0.991
P3	Paclitaxel	0.136	0.906	0.461	0.927

**Table 3:** Percentage of cumulative drug release after 12 weeks of incubation under physiological conditions (PBS, 37 °C).

Formulation No	Sirolimus (%)	Tacrolimus (%)	Paclitaxel (%)
1	90.93	90.93	40.82
2	54.51	76.31	34.62
3	55.11	98.50	50.44
4	42.01	90.93	44.73
5	28.03	89.95	46.10
6	22.60	74.47	53.19
7	25.07	86.96	67.65
8	23.20	84.36	61.20
9	19.31	92.90	60.13
10	21.79	84.78	51.94
11	17.53	70.93	39.80
12	21.70	91.56	48.20

## Data Availability

The original contributions presented in this study are included in the article and Supplementary Material. Further inquiries can be directed to the corresponding author.

## Supplementary Materials

The following supporting information can be downloaded at: https://www.mdpi.com/article/10.3390/ijms27083695/s1.
