# Supplementary material for: Biodegradable Polymer-Coated Surgical Sutures for Controlled and Sustained Release of Sirolimus, Tacrolimus, and Paclitaxel
Source: Int J Mol Sci. 2026 Apr 21;27(8):3695. doi: 10.3390/ijms27083695 (PMC13115729; doi:10.3390/ijms27083695)
Supplement: Supplementary file 1 [file ijms-27-03695-s001.zip › ijms-4230932-supplementary.pdf]

The Supplementary Materials include detailed kinetic modeling parameters (Table S1) for all investigated formulations

Table S1. Kinetic modeling parameters for all formulations. kH, Higuchi release constant; n, release exponent in the Korsmeyer–Peppas model. Higuchi fitting was performed using the full release profile, whereas Korsmeyer–Peppas fitting was performed for the initial release phase ($M_t/M_\infty \leq 0.6$).

Formulation	Higuchi kH	Higuchi R^2	Peppas n	Peppas R^2
**S1**	0.299	0.981	0.806	0.998
**S2**	0.194	0.998	0.761	0.991
**S3**	0.149	0.906	0.461	0.927
**S4**	0.138	0.981	0.636	0.974
**S5**	0.092	0.981	0.636	0.974
**S6**	0.080	0.998	0.761	0.991
**S7**	0.082	0.981	0.636	0.974
**S8**	0.076	0.981	0.636	0.974
**S9**	0.052	0.906	0.461	0.927
**S10**	0.072	0.981	0.636	0.974
**S11**	0.062	0.998	0.761	0.991
**S12**	0.059	0.906	0.461	0.927
**T1**	0.299	0.981	0.806	0.998
**T2**	0.271	0.998	0.831	0.997
**T3**	0.266	0.906	0.770	1.000
**T4**	0.299	0.981	0.806	0.998
**T5**	0.296	0.981	0.806	0.998
**T6**	0.265	0.998	0.831	0.997
**T7**	0.286	0.981	0.806	0.998
**T8**	0.277	0.981	0.781	0.996
**T9**	0.251	0.906	0.769	1.000
**T10**	0.279	0.981	0.781	0.996
**T11**	0.252	0.998	0.816	0.996
**T12**	0.248	0.906	0.770	1.000
**P1**	0.134	0.981	0.636	0.974
**P2**	0.123	0.998	0.761	0.991
**P3**	0.136	0.906	0.461	0.927
**P4**	0.147	0.981	0.636	0.974
**P5**	0.152	0.981	0.636	0.974
**P6**	0.189	0.998	0.761	0.991
**P7**	0.222	0.981	0.712	0.989
**P8**	0.201	0.981	0.654	0.979
**P9**	0.163	0.906	0.481	0.938
**P10**	0.171	0.981	0.636	0.974
**P11**	0.142	0.998	0.761	0.991
**P12**	0.130	0.906	0.461	0.927
